# Neurons as a model system for cryo-electron tomography

**DOI:** 10.1016/j.yjsbx.2022.100067

**Published:** 2022-03-09

**Authors:** Benoît Zuber, Vladan Lučić

**Affiliations:** a Institute of Anatomy, University of Bern, Bern 3012, Switzerland; b Max-Planck-Institute of Biochemistry, Am Klopferspitz 18, 82152 Martinsried, Germany

**Keywords:** Neurons, Synapses, Electron microscopy, Cryo-electron tomography

## Abstract

Cryo-electron tomography (Cryo-ET) provides unique opportunities to image cellular components at high resolution in their native state and environment. While many different cell types were investigated by cryo-ET, here we review application to neurons. We show that neurons are a versatile system that can be used to investigate general cellular components such as the cytoskeleton and membrane-bound organelles, in addition to neuron-specific processes such as synaptic transmission. Furthermore, the synapse provides a rich environment for the development of cryo-ET image processing tools suitable to elucidate the functional and spatial organization of compositionally and morphologically heterogeneous macromolecular complexes involved in biochemical signaling cascades, within their native, crowded cellular environments.

## Highlights

• Cryo-ET imaging of neurons is a versatile system for cell biology in situ.
• Structural and spatial localization analysis yields new insights into synaptic transmission.
• The synapse provides a rich environment for the development of image processing tools.

## Graphical abstract

**Figure:**
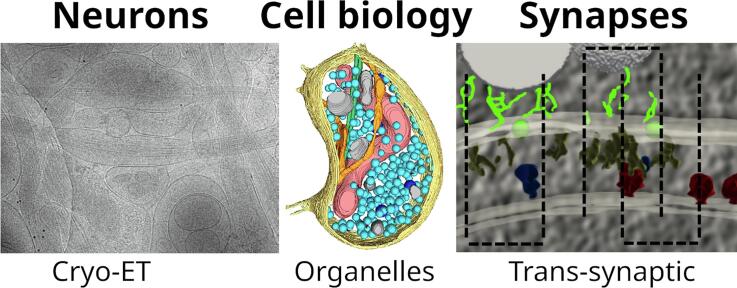

## 1 Introduction

From the early days of cryo--electron tomography (cryo-ET), it was recognized that its main potential lies in the ability to investigate pleomorphic protein assemblies, organelles and cells in their native state (Baumeister et al., 1999). This arises from the combination of the molecular-level preservation by sample vitrification with the high resolving power of electron microscopy, which makes cryo-ET uniquely suited to elucidate the functional organization of cellular components in situ.

Because electrons strongly interact with matter, sample thickness is a fundamental constraint in all modalities of transmission electron microscopy (TEM), including cryo-ET. The maximal usable thickness increases with the electron acceleration voltage because of the increased mean free electron path. At 300 V, the highest voltage routinely used for cryo-ET, cellular samples up to 500 nm in thickness are imaged. However, imaging thick samples by TEM suffers from multiple electron scattering-induced decrease of the number of electrons that contribute to the image formation (Lucic et al., 2005). Tomogram reconstruction and image processing are further aggravated because in TEM, images are essentially projections through the sample and because in TEM images of thick samples, the structural information from different sample depths overlaps. Consequently, thinner samples allow reaching higher resolution, leading to the preferred sample thickness of up to 200–300 nm.

Archaea and bacteria were investigated by cryo-ET because of their relatively small size (Grimm et al., 1998, MMilne and Subramaniamilne and Subramaniam, 2009, Tocheva et al., 2010). In eukaryotes, nucleus is the largest organelle, it is responsible for storing chromosomes and transcription. Most eukaryotic cell culture models have nuclei of several micrometers in diameter, rendering most of the volume of intact cells inaccessible to cryo-ET because of the high thickness. Some eukaryotic cells possess regions far from the nucleus that are thin enough for cryo-ET. These peripheral regions are mostly free of membrane-bound organelles and often contain a specially organized cytoskeleton, such as in lamellipodia, as evidenced by the first intact cell cryo-ET study (Medalia et al., 2002). In order to expand the applicability of cryo-ET to the cell interior, different methods for thinning vitrified cells were developed. However, these methods add another layer of difficulty because they are either resolution-limiting (e.g. CEMOVIS), disruptive (e.g. unroofing), or are in most cases low-throughput (cryo-focused ion beam milling) (Al-Amoudi et al., 2004, Marko et al., 2007, Schaffer et al., 2017). While automation of cryo-focused ion beam (cryo-FIB) milling makes a big step towards its routine use, it still results in an order of magnitude smaller sample area suitable for cryo-ET than what a thin sample can provide, which is limiting for some, but not all applications (Klumpe et al., 2021).

**Fig. 1:**
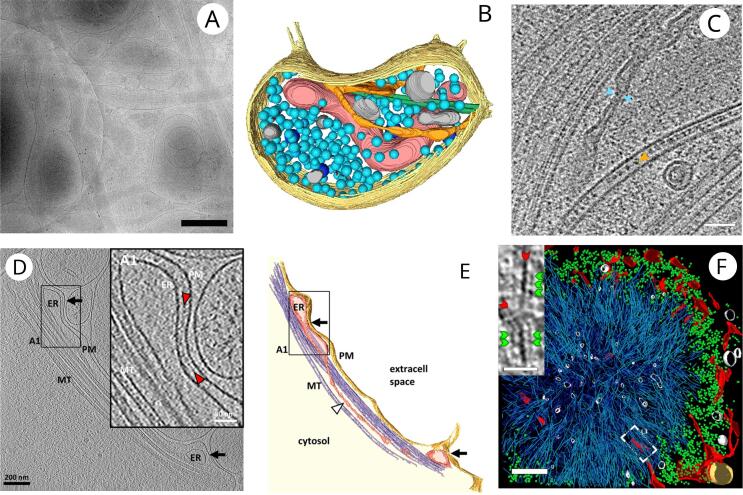
Cell-ubiquitous structures observed in neurons. (A) Low-magnification cryo-EM image of a dissociated neuronal culture (see Box 1) grown on EM grid (bar, 500 nm). (B) 3D segmentation of a bouton from a dissociated neuronal culture containing plasma membrane (yellow), mitochondrion (red), microtubules (green), SER (orange), synaptic vesicles (light blue), dense core vesicles (dark blue) and other membranous compartments (grey). (C) A tomographic slice through an axon in human brain organoid. It contains several microtubules. In one of them, intraluminal particles are particularly evident. ER with an extremely narrow portion is marked with blue arrowheads and microtubule lumenal density by a orange arrowhead (bar, 50 nm). (D) Tomographic slice of an ER-plasma membrane contact sites (black arrows) in cultured neurons (bar, 200 nm). Inset. Higher-magnification tomographic slice of the ER–plasma membrane contact in the labeled region. The intermediate density between the ER and the plasma membrane is indicated by red arrowheads (bar, 50 nm). (E) 3D rendering of the tomogram depicted in D. (F) 3D rendering of an inclusion body in a neuron transfected with polyQ-huntingtin. ER membranes (red), polyQ-huntingtin fibrils (cyan), ribosomes (green), vesicles (white), and mitochondria (gold) (bar, 400 nm). Inset. Magnified view of a tomographic slice corresponding to the area labeled with a white rectangle showing polyQ-huntigntin fibrils (red arrowheads) decorated with globular densities (green arrowhead) (bar, 30 nm). Images are reproduced with permission from (Schrod et al., 2018) (A, B), (Hoffmann et al., 2021) (C), (Fernández-Busnadiego et al., 2015) (D, E) and (Bäuerlein et al., 2017) (F).

Neurons are characterized by large cellular processes, axons and dendrites. The volume of a neuron consists almost entirely of its axon and dendrites, while the volume of the cell body, i.e the region surrounding the nucleus, is negligible (Braitenberg, 2001). While axons and dendrites can form a highly interwoven thick layer, they are often sufficiently thin for cryo-ET observations without the need for thinning. Hence, imaging intact neurons provides access to organelles and protein complexes that are ubiquitous to many cell types, whereas these structures may not be observed in other cell culture models without thinning (Fig. 1A, B).

Neuronal synapses are specialized junctions between neurons and effector cells that can transmit a presynaptic signal to the postsynaptic cell. Synaptic complexes are known for their exquisite time precision and complex signaling cascades, making the elucidation of the function and structure of these complexes in synaptic transmission a rich and long-standing research topic.

Here we review research focused on cryo-ET of neuronal samples (see Box 1). We discuss biological findings that are specific to neurons, as well as those that have a broader relevance in cell biology. Furthermore, we review method developments that benefited from neuronal samples, such as cryo-correlative microscopy, as well as image processing methods that are generally applicable to in situ cryo-ET. Electron tomography studies of synapses prepared by methods involving chemical fixation and dehydration were recently reviewed elsewhere (Liu et al., 2019, Zuber and Lucic, 2019). As this review aims to provide an unbiased assessment of the current literature, the pioneering research and eminence of the Baumeister lab in this field is made evident by the large number of papers reviewed here originating from this lab.

**Box 1. Neuronal preparations.**

**Organotypic neuronal cultures.**Brain slices, typically obtained from adult rodent hippocampi, are grown in culture for a period between one and few weeks. After vitrification, slices need to be thinned for cryo-ET imaging. So far, the thinning was done only using CEMOVIS (Al-Amoudi et al., 2004, Zuber et al., 2005, Fernández-Busnadiego et al., 2010).

**Dissociated neuronal cultures.** Neurons dissociated from the brain region of interest are grown on electron microscopy dishes for few days if only axons are investigated, or at 10–21 days for synapses. Axons, dendrites and synapses can be imaged in cryo-ET directly (Lucic et al., 2007), but imaging dense cultures requires thinning by focused ion beam under cryo conditions (cryo-FIB) (Marko et al., 2007, Schaffer et al., 2017), and may require an additional processing step to remove contamination-induced 3D reconstruction artefacts (Fernandez et al., 2016). Imaging cell body requires thinning by cryo-FIB (Bäuerlein et al., 2017).

**Cell culture unroofing**. Mechanical or detergent-based disruption of the “apical” cell membrane and removal of the bulk of cell growing on EM grid, leaving only the parts of the cells that are directly or indirectly connected to the membrane attached to the substrate grid (Peitsch et al., 2016).

**Synaptosomal fraction.** Cellular fraction that can be obtained from different brain parts. It contains synaptosomes, that is plasma membrane-enclosed pre- and postsynaptic terminals that are attached to each other but detached form the cell bodies. Both terminals retain an important part of their function (Nicholls and Sihra, 1986, Martinez-Sanchez et al., 2021). Does not require thinning for cryo-ET.

**Postsynaptic density fraction.** Detergent extracted fraction from synaptosomes (Cohen et al., 1977).

## 2 Cell-ubiquitous components visualized in neurons

Among the many organelles and subcellular structures that have been investigated by cryo-ET, cytoskeleton elements have been a prime target. Their filamentous nature makes them easily identifiable and distinguishing between microtubules, intermediate filaments, and actin filaments, based on their ultrastructure is straightforward. Many aspects of cytoskeleton structure and function relationship have been investigated. In particular, an early contribution of neuronal cryo-ET was the observation that neuronal microtubules contain dense intraluminal particles (Garvalov et al., 2006). These finding were further elaborated in neuronal axons and fibroblasts, cells that like neurons contain large thin protrusions, confirming that early observations done in chemically fixed and resin-embedded neurons were not artefactual (Fig. 1C) (Bouchet-Marquis et al., 2007, Fukuda et al., 2015, Schrod et al., 2018, Chakraborty et al., 2020, Rodríguez Echandía et al., 1968, Hoffmann et al., 2021). For a long time, the composition and function of the microtubular intraluminal particles remained unknown. A recent in vitro cryo-ET publication has brought strong evidence that microtubule-associated protein 6 (MAP6, also known as stable tubule-only peptide), a large protein know for providing stability to microtubules is one of the main components of the intralumenal particles and shed light on their function (Cuveillier et al., 2020). MAP6 is an intrinsically disordered protein and it was the first neuronal microtubule-associated protein to be discovered (Cuveillier et al., 2021). It stabilizes neuronal microtubules, and can interact with membranes, as well as with actin. MAP6 knock-out in mice affects synaptic plasticity and leads to schizophrenia-like phenotypes. It was also shown that MAP6 indeed stabilizes microtubules, promotes their growth, increases their curvature, and forms apertures in the microtubule lattice, possibly relieving mechanical stress, consistent with the morphology and function of microtubules in neurons (Cuveillier et al., 2020).

The resolution that can be now routinely obtained by cryo-ET enables the determination of the microtubule polarity. It was shown that the vast majority of microtubules in growing axons from brain organoids have their plus-end directed towards the periphery (Hoffmann et al., 2021). This is in agreement with previous studies showing that microtubules have the same polarity in axons and mixed polarity in dendrites, which serves to separate axonal and dendritic cargo trafficking, as kinesins move towards the plus end dyneins towards the minus end (van Beuningen and Hoogenraad, 2016). While this was confirmed in axons of cultured mouse neurons, it was reported that 32% of axons in *Drosophila* primary culture contained microtubules of mixed polarity (Foster et al., 2022). Furthermore, whereas virtually all microtubules were formed of 13 protofilaments in mouse neurons, about half of the microtubules in*Drosophila* consisted of 12 protofilaments and the other half of 13 protofilaments.

Intermediate filaments are another cytoskeletal elements that has been visualized by cryo-ET in the skin, in neurons and in glial cells after cryo-sectioning (Norlén et al., 2007; Zuber, unpublished). The molecular architecture of the nuclear intermediate filaments, the lamins, was found to form a scaffold below the nuclear envelope in unroofed fibroblasts (see Box 1) as well as in cryo-FIB lamellae (Turgay et al., 2017, Kronenberg-Tenga et al., 2021). Such a level of detail had never been visualized in plastic-embedded samples. Recently, loosely packed cytoskeletal arrays were revealed in mammalian dorsal root ganglion axons, containing intermediate filaments, microtubules, actin, and an unknown type of thin filaments, which the authors hypothesized were made by spectrins (Foster et al., 2022).

A wealth of in situ structural information of actin filaments has been obtained from cryo-ET datasets. This quest started in Prof. Baumeister’s department in thin slime mold Dyctiostelium discoideum (Medalia et al., 2002). The arrangement of actin was then characterized in neuronal axons, the pre- and the postsynapse, as well as in fibroblasts (Koning et al., 2008, Fernández-Busnadiego et al., 2010, Fernández-Busnadiego et al., 2011, Fukuda et al., 2015, Schrod et al., 2018, Hoffmann et al., 2021, Foster et al., 2022). Interestingly, actin filaments in axons were in all cases reported to run along the axon, parallel to microtubules. Tunelling nanotubes are actin-containing structures that can transport organelles between two cells. The detailed arrangement of actin and transported organelles in those nanotubes was characterized by cryo-ET of a neuronal tumor cell line (Sartori-Rupp et al., 2019).

The understanding of the architecture of organelles has advanced thanks to the application of cryo-ET to intact neurons. In addition to synaptic vesicles and endocytic vesicles, mitochondria, endosomes, and the endoplasmic reticulum (ER) have been characterized in detail (Fernández-Busnadiego et al., 2015, Schrod et al., 2018, Fischer et al., 2018, Hoffmann et al., 2021, Foster et al., 2022). In particular cryo-ET showed that in the native state, the mitochondria and ER in axons and dendrites can adopt a very thin shape (Fig. 1C). The ER membrane shows strong surface discontinuities, or sharp kinks. that was routinely observed in resin-embedded samples but it was not clear if the angular aspect of the ER membrane was artifactual.

The power of cryo-ET lies in its ability to image individual, pleomorphic proteins and protein complexes. It was demonstrated that a wide variety of membranous organelles are organized via pleomorphic linkers, including vesicles that are engulfed within multivesicular bodies and two different types of ER-plasma membrane contacts likely composed of extended-synaptotagmins and stromal interaction molecule (Fig. 1D, E) (Fernández-Busnadiego et al., 2015, Schrod et al., 2018). Cryo-ET has revealed in numerous instances the pleomorphic linker-mediated interactions between microtubules and organelles being transported in neurons, such as transport vesicles, synaptic vesicle packets and ER (Schrod et al., 2018, Sartori-Rupp et al., 2019, Hoffmann et al., 2021, Foster et al., 2022). Thanks to the recent instrumentation improvements, a sufficient resolution was reached to visually identify the dynein/dynactin complex as the linker connecting a microtubule to a transport vesicle, the transport vesicle-bound V-ATPase, as well as the 26S proteasome (Asano et al., 2015, Foster et al., 2022).

Neurodegenerative disorders, a major public health concern, all have misfolded protein accumulation and neuronal cell death in common. PolyQ and poly-GA inclusions are hallmarks of several neurodegenerative disorders. PolyQ inclusions were shown to consist of amyloid-like fibrils, which interact with the ER, deform and disorganize it (Fig. 1F) (Bäuerlein et al., 2017). Poly-GA inclusions on the other hand consisted of densely packed twisted ribbons (Guo et al., 2018). They were bound to numerous 26S proteasome complexes, suggesting that these aggregates may disrupt proteostasis by reducing the availability of proteasome. This research proposes a possible mechanisms underlying the cellular pathology of polyQ and poly-GA inclusions. As the inclusions are several micrometers in diameter, cryo-FIB milling of neurons and cell-lines was necessary to obtain a few hundreds nanometer-thick lamella.

Some viruses, such as herpesvirus, hijack microtubule-based transport and the synaptic exocytosis machinery to propagate. Early cryo-ET research found that non-enveloped capsids were transported along axonal microtubules, some of them containing DNA, whereas others were empty (Ibiricu et al., 2011). Some capsids were enveloped in the mid-axon, while most capsids became enveloped at presynaptic terminals. Recently, capturing a virus undergoing exocytosis showed that the process is biphasic, comprising a fast membrane fusion step and a slow phase of fused membrane flattening (Liu et al., 2020).

## 3 Neurons are particularly suitable for cryo-correlative microscopy

Labelling specific intracellular proteins with probes that are visible in cryo-EM is notoriously difficult. Fluorescent labels, on the other hand, are the daily bread of cell biologists. Yet the resolution of fluorescence microscopy is orders of magnitude too low for assigning protein identity to a particular molecule visualized in cryo-ET. Great hopes lie in the development of super-resolution cryo-CLEM, which have the potential to correlate cryo-ET structures with molecular resolution localization by super-resolution cryo-fluorescence microscopy. While heating due to intense laser illumination is a serious problem that can result in sample devitrification, very promising results were recently achieved (Moser et al., 2019).

The well defined shape of axons makes the application of correlative light and electron microscopy (CLEM) particularly suitable. As the search for a structure-of-interest in cryo-ET can be very slow, the ability to pin down the cell regions containing the structure-of-interest by fluorescence microscopy and navigating to the same area for cryo-ET imaging results in a massive productivity increase. One of the two pioneering cryo-CLEM holders was developed in the Baumeister department and tested on a neuronal cell line (Sartori et al., 2007, Schwartz et al., 2007). Neuronal cultures were used to demonstrate the power of a recently developed high-vacuum optical cryo-stage (Li et al., 2018).

On the application side, neuronal axons and dendrites were unequivocally distinguished by correlating fluorescently tagged markers MAP2 and SMI312 to cryo-ET (Hoffmann et al., 2021). Excitatory and inhibitory synapses were formally indentified by fluorescently tagging the postsynaptic scaffold proteins PSD-95 and gephyrin, respectively (Tao et al., 2018). Correlating their cryo-fluorescence images with cryo-electron tomograms, the authors demonstrated that excitatory synapses have a thicker postsynaptic density than inhibitory synapses, in agreement with the picture obtained from resin-embedded samples. However, inhibitory and excitatory synapses were found to have undistinguishable cleft width, whereas resin-embedded samples showed a thinner cleft in inhibitory synapses.

## 4 Synapses: neuron-specific structure and a model system for image processing

Neuronal synapses are characterized by a close apposition between the presynaptic and the postsynaptic terminal. This organization allows directed transmission of the presynaptic signal to the postsynaptic side. We will focus on chemical synapses, where synaptic transmission is mediated by the neurotransmitter release from the presynapse and its binding to the postsynaptic receptors. The neurotransmitter molecules reach the postsynapse by diffusion through the extracellular region between the synaptic terminals, the synaptic cleft.

Among the three above-mentioned synapse parts, the synaptic cleft is arguably the least understood, both molecularly and functionally (Biederer et al., 2017). It was clear from the first observations of vitrified synapses imaged by TEM of organototypic neuronal cultures and by cryo-ET of isolated synapses (synaptosomes, see Box 1) that synaptic adhesion complexes are extensively laterally connected (Zuber et al., 2005, Lucic et al., 2005). Furthermore, an increased density was observed in the central cleft layer that is positioned roughly parallel to the pre- and postsynaptic plasma membranes, leading to the proposal that synaptic adhesion complexes form a network-like structure. This is in contrast to the current textbook view based on protein interaction data, where synaptic adhesion complexes mostly bind in trans, leading to the picture where simple molecular bridges between the pre- and postsynaptic terminals dominate the molecular organization in the synaptic cleft. More recently, a work that combined cryo-ET with genetic manipulations and super-resolution imaging showed that SynCAM1, one of the more prominent synaptic adhesion proteins, which is known to drive synaptic assembly, is primarily localized along the edge of the synaptic cleft and forms dynamic nano-domains (Perez de Arce et al., 2015).

**Fig. 2:**
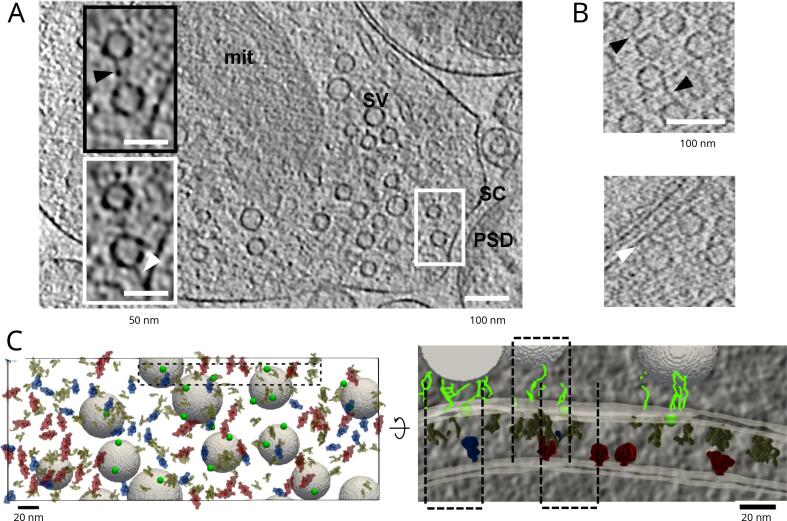
Cryo-ET of central nervous system synapses. (A) Synapses from synaptosomal cellular fraction, insets show a magnified view of a connector (above) and tether (below). PSD denotes the postsynaptic density, SC synaptic cleft, SV synaptic vesicles and mit mitochondrion. (B) Synapses from dissociated neuronal cultures. White arrowheads point to tethers and black to connectors (A and B). (C) Mapping synaptic complexes, view from the postsynaptic side (left). Tripartite trans-synaptic complexes (indicated by dashed lines), magnified view of the inset from the left panel, view from the cleft side (right). Presynaptic complexes are shown in yellow, putative AMPA receptors in blue) putative NMDA receptors in red and synaptic vesicle tethers in green (circles indicate centroids of tether clusters and lines individual tethers). Images are reproduced with permission from (Fernández-Busnadiego et al., 2010) (A) and (Martinez-Sanchez et al., 2021) (B and C).

The presynaptic terminal can contain hundreds of synaptic vesicles organized in functionally defined pools (Rizzoli and Betz, 2005). While the existence of synaptic vesicle-bound filaments was described before, the full 3D visualizations of presynaptic terminals by cryo-ET of synptosomes and organotypic cultures (see Box 1) unequivocally established that filaments that interlink synaptic vesicles and tether them to the plasma membrane are the most prominent structures that organize the vesicles (Zuber and Lucic, 2019). Tethers and connectors were observed in all synaptic preparation investigated so far; in organotypic and dissociated neuronal cultures, and synaptosomes, as well as in non-synaptic axonal boutons, that is in presynaptic terminals that do not form a junction with a postsynaptic terminal (Fig. 2A, B). Similar structures were also detected on vesicles involved in transport along microtubules and internalized in endosomes, arguing that tether- and connector-like structures may have a wide range of cellular roles (Schrod et al., 2018).

Quantitative characterization of various properties and localization of tethers and connectors in wild-type synapses imaged by cryo-ET set a stage for exploring genetically and pharmacologically perturbed synapses, in order to investigate their function and molecular properties. Results from a series of cryo-ET investigations showed that the number and length of tethers determines the synaptic vesicle progression towards the neurotransmitter release (Fernández-Busnadiego et al., 2010). It is likely that multiply tethered SVs constitute the structural correlate of the readily releasable pool, that is synaptic vesicles that are functionally prepared for the release, and that the short tethers are particularly important for these vesicles. It was also shown that RIM1*α* protein is important for tether formation or maintenance and that other RIM isoforms may provide a partial compensation when RIM1*α* is genetically ablated (Fernández-Busnadiego et al., 2013). Subsequent electro-physiological experiments confirmed that tethering deficits observed by cryo-ET directly correspond to the functional state of the presynaptic terminal.

Furthermore, synaptic vesicle connectors were shown to be distributed throughout the region of the presynaptic terminal and to organize the majority of vesicles in large clusters at the resting state (Fernández-Busnadiego et al., 2010). They are dynamic structures that respond to stimulation and decrease of dephosphorylation by reducing the overall vesicle connectivity, which might increase their availability for release. Interestingly, *α*-synuclein, a small protein that is causally linked to the pathogenesis of Parkinson’s disease, was shown to support vesicle connectivity but also to interfere with tethering (Vargas et al., 2017). Taken together with the data from synapses lacking RIM1*α* (Fernández-Busnadiego et al., 2013), it can be argued that connectors modulate neurotransmitter release.

Both tethers and connectors are pleomorphic structures that have a wide distribution of lengths. The resulting structural heterogeneity precludes the use of standard image processing methods such as template matching and subtomogram averaging (Förster et al., 2010, Wan and Briggs, 2016). The software package Pyto was specifically developed for the detection and analysis of structurally heterogeneous, membrane-bound complexes in cryo-electron tomograms (Lucic et al., 2016), and was used to obtain the results presented earlier in this section. This package detects tethers and connectors in a fully automated manner, based on the hierarchical connectivity segmentation. In short, connected clusters of pixels that contact two predefined boundaries (two different vesicles membranes for connectors, and a vesicle and the plasma membrane for tethers) are selected at a given greyscale level (pixel value). To account for the variable background and a high noise level in cellular cryo-tomograms, this task is repeated for multiple greyscale levels yielding a hierarchical organization of the selected clusters, from which a set of non-overlapping segments (connectors and tethers) is extracted. Furthermore, morphological, greyscale and topological properties of connectors and tethers, as well as their localization and inter-relation are determined. Finally, the Pyto package allows organizing the data in biologically relevant experimental groups and performing statistical analysis and inference. Considering the amount, diversity and variability of synaptic cryo-ET data, the detection - analysis pipeline implemented in Pyto was critical for the development of the structural model of neurotransmitter release (Lucic et al., 2016).

Cryo-ET imaging of synapses from dissociated neuronal cultures is complicated by the opposing requirements to grow the cultures that are thin enough for TEM, yet sufficiently dense to develop synapses, and it is further aggravated by the inherent variability of this preparation (Lucic et al., 2007, Schrod et al., 2018). Nevertheless, in the exemplary show of skills, hundreds of synapses from dissociated hippocampal neuronal cultures (see Box 1) were imaged by cryo-ET (Tao et al., 2018, Tao et al., 2018, Liu et al., 2020). It was shown that chronic inactivity leads to the accumulation of dense core vesicles (Tao et al., 2018), medium size transport vesicles known to carry proteins required for the presynaptic terminal assembly. Dense core vesicles were also characterized by cryo-ET in immature neurons (Schrod et al., 2018).

The postsynaptic density of excitatory synapses from neuronal cultures, a thick intracellular layer apposed to the presynaptic membrane, was shown to contain a complex network of short filaments (Tao et al., 2018), as previously visualized in synaptosomes (Fernández-Busnadiego et al., 2011). Similar structures, described as a collection of small filamentous and globular proteins, were detected in cryo-ET images of detergent-extracted postsynaptic density fraction (see Box 1) (Farley et al., 2015). The postsynaptic density of inhibitory synapses was found to consist of a single protein layer located at a short distance, parallel to the postsynaptic membrane (Tao et al., 2018). Recently, a template-free approach, whereby particles were picked by hexagonal spatial sampling on the postsynaptic membrane of inhibitory synapses and then subjected to the standard subtomogram 3D classification and refinement, led to in situ average densities that likely represent GABA_A_receptors. Further processing resolved their postsynaptic binding partners, likely gephryn proteins. In addition, the spatial organization analysis revealed that GABA_A_receptors tend to associate with each other in groups of two or three receptors, and also to form larger well-defined assemblies (Liu et al., 2020).

Another, more complex template-free procedure was recently implemented in the PySeg package, where Discrete Morse theory-based tracing of biological material is used to detect membrane-bound protein complexes, which are then classified by Affinity propagation clustering (Martinez-Sanchez et al., 2020). This procedure was shown to successfully classify complexes in biological systems containing hundreds of different protein species into classes that contain morphologically similar complexes. Namely, while some of these classes were heterogeneous, comprising mixtures of different complexes, other were homogeneous, that is comprising mostly one specific complex type. Hence, the homogeneous classes are suitable for further standard subtomogram processing, which resulted in de novo average densities of complexes as small as 200 kDa. An application of this detection – classification procedure yielded first de novo average structures of postsynaptic ionotropic glutamate receptors (iGluRs) in their physiological composition within their native environment of interacting proteins and lipids (Martinez-Sanchez et al., 2021).

In addition to discriminating between different types of complexes, the detection-classification procedure yields a precise position for each complex. This allowed mapping presynaptic and postsynaptic complexes to their exact locations at the synapse (Fig. 2C) (Martinez-Sanchez et al., 2021). The subsequent spatial organization analysis of pre- and postsynaptic complexes showed that tripartite trans-synaptic complexes provide a structural link between synaptic vesicles and postsynaptic receptors. This finding might explain the precise alignment of neurotransmitter release sites and neuroreceptors required for efficient synaptic transmission (Nair et al., 2013, Tang et al., 2016, Biederer et al., 2017, Chen et al., 2018). Importantly, it was found that the tripartite trans-synaptic complexes combine in a non-uniform manner to form larger molecular assemblies. The size of these large assemblies is consistent with the trans-synaptic nanocolumns previously observed with super-resolution fluorescence microscopy, arguing that they are important for synaptic transmission.

The computational approaches mentioned above have different goals. Pyto package provides a straightforward detection of pleomorphic complexes that link two different membranes, their quantitative characterization and the statistical analysis between different experimental conditions (Lucic et al., 2016). The approach of (Liu et al., 2020) was used to determine the average density of a dominant complex in a particular region, using standard subtomogram averaging tools. Because PySeg employs more advanced density tracing and classification, it is suitable for averaging multiple types of membrane-bound complexes that are randomly distributed in the same cellular region (Martinez-Sanchez et al., 2020). All three approaches address spatial organization of detected complexes, Pyto in relation to a cellular structure such as plasma membrane, while the other two are used to characterize the distribution of complexes based on Voronoi entropy (Liu et al., 2020) and Ripley’s functions (Martinez-Sanchez et al., 2021, Martinez-Sanchez et al., 2022).

## 5 Discussion

Despite the fact that neurons are currently far from being among the most often investigated cellular systems by cryo-ET, the current literature shows that multiple aspects of high-resolution, in situ imaging can be addressed by cryo-ET of neuron-related preparations.

The functioning of human brain is arguably the most distinct characteristic of our species. Accordingly, neuronal cells have many special morphological and functional characteristics, the elucidation of which makes important and complex research targets. As the contact point between neurons, which are know for their high inter-connectivity, the synapse is a prime example. Recent work detected complexes that traverse the synapse, linking synaptic vesicles with postsynaptic receptors, and showed that they form large non-uniform trans-synaptic assemblies. Furthermore, cryo-ET investigations provided a full 3D detection and quantitative characterization of synaptic vesicle tethers and connectors, synaptic complexes that were previously not well understood, which strongly implicated them in neurotransmitter release. Importantly, cryo-ET led to de novo average densities of native ionotropic receptor complexes, despite the large number of different protein species present at synapses.

In order to form synapses and maintain their function, proteins and mRNA need to be transported over large distances, from the cell body, along axons and dendrites (Hirokawa and Takemura, 2005). The underlying process, microtubules-based transport, occurs in both directions and is common to other cell types, but the distances that axonal cargo has to traverse are much larger in neurons, placing additional requirements. Cryo-ET imaging of axonal transport identified different types of transport vesicles, detected their cargo and observed synaptic vesicles transport packets. Cryo-ET is likely to be key in the pursuit of the molecular and biophysical mechanism at their origin. Furthermore, it is an ideal method to investigate the structure and function relation of contacts between organelles, which have recently been recognized as key players for the function of cells (Prinz et al., 2020).

Neurons are also suitable for investigations of basic cell-biological processes by cryo-ET. For example, imaging cytoskeleton in neuronal cultures led to in situ structural information and organization of microtubules, intermediate filaments and actin filaments. Cryo-ET of neurons offers an ideal platform to obtain a quantitative morphological characterization of ER and to study the fine architecture of misfolded protein inclusions involved in neuro-degenerative disorders.

In comparison with other cellular systems, neurons have the advantages that they are native cells that can be grown in primary cultures obtained from mammals and that large parts of neurons can be imaged by cryo-ET without thinning. Neuronal cultures require careful handling and are more difficult to grow than other standard cell cultures, especially when grown on electron microscopy grids and expected to reach the synaptogenesis stage (Kaech and Banker, 2006). Similar cells, such as fibroblasts, are also used because they are easier to culture. Nevertheless, neuronal cultures provide a highly relevant cellular system for human physiology and disease that is suitable for cryo-ET.

From a larger, cell-biological perspective, neuronal synapses can be regarded as a system that combines precise and complex biochemical cascades with lipid membrane dynamics. The synapse provides a rich environment for the development of cryo-ET image processing routines for arguably the most common type of biological systems, crowded environments characterized by a large number of molecular species forming pleomorphic complexes that do not form periodic structures and cannot be visually identified. The procedures for template-free detection, classification, quantitative characterization and spatial analysis of compositionally and morphologically heterogeneous membrane bound complexes, Pyto and PySeg, were created with the goal of applications to synapses, but the core procedures were developed system-agnostic. Therefore they are suitable for applications to other biochemical signaling-rich environments and cell junctions, such as those involved in immune response and development, where precise spatial organization of different molecular complexes plays a key role.

In conclusion, neurons provide a versatile biological system for addressing multiple aspects relevant for cellular cryo-ET, from in situ imaging of cellular components and cryo-correlative approaches to the unbiased detection, molecular identification, structure determination and spatial analysis of complexes embedded in molecularly heterogeneous environments.

## CRediT authorship contribution statement

**Benoît Zuber:** Conceptualization, Writing. **Vladan Luči**ć**:** Conceptualization, Writing.

## Declaration of Competing Interest

The authors declare that they have no known competing financial interests or personal relationships that could have appeared to influence the work reported in this paper.
